# Phyllostachyos Caulis in Taeniam extract stimulates longitudinal bone growth via IGF-1 and JAK2/STAT5 signaling in rats

**DOI:** 10.1371/journal.pone.0331834

**Published:** 2025-09-30

**Authors:** Yongwun An, Hee Woo Lee, Yun Chan Jung, Hyun Jae Woo, Younghoon Kim, Hohyun Kim, Yong Joon Jeong, A. M. Abd EI-Aty, Ji Hoon Jeong

**Affiliations:** 1 Department of Pharmacology, College of Medicine, Chung-Ang University, Seoul, Republic of Korea; 2 Korea Medicine Research Institute, Inc., Seongnam, Republic of Korea; 3 Chaon Co., Ltd., Yongin, Republic of Korea; 4 Department of Pharmacology, Faculty of Veterinary Medicine, Cairo University, Giza, Egypt; 5 Department of Medical Pharmacology, Medical Faculty, Ataturk University, Erzurum, Turkey; 6 Department of Global Innovative Drugs, Graduate School of Chung-Ang University, Seoul, Republic of Korea; Kenyatta University School of Pure and Applied Sciences, KENYA

## Abstract

Longitudinal bone growth, which is regulated by endocrine and paracrine factors, is a critical determinant of linear growth during childhood. This study investigated the effects of an aqueous extract of Phyllostachyos Caulis in Taeniam (PCE) on longitudinal bone growth and its regulatory effects on circulating insulin-like growth factor-1 (IGF-1) in rats. Twenty-eight adolescent rats were randomly assigned to four groups (n = 7 per group): control, recombinant human growth hormone (rhGH) 20 μg/kg, PCE 200 mg/kg, and 400 mg/kg. After 10 days of administration, the serum levels of growth hormone (GH), IGF-1, IGF binding protein 3 (IGFBP3), and osteocalcin, as well as tibial length, were measured. In addition, the mRNA expression levels of IGF-1 and IGFBP3 in liver tissue were quantified via real-time polymerase chain reaction (qRT-PCR), and the protein expression levels of Janus kinase 2 (JAK2), signal transducer and activator of transcription 5 (STAT5), and IGF-1 were assessed via western blotting. Compared with the control, 400 mg/kg PCE significantly increased the serum levels of GH, IGF-1, IGFBP3, and osteocalcin in rats by 486.6%, 73.7%, 22.5%, and 27.8%, respectively (*P* < 0.01), and increased the tibial length by 7.4% (*P* < 0.01). Compared with the control, 400 mg/kg PCE also increased the mRNA expression of IGF-1 and IGFBP3 in the liver by 5.2-fold (*P* < 0.01) and 7.3-fold (*P* < 0.05), respectively. Moreover, compared with the control, 400 mg/kg PCE increased hepatic IGF-1 protein expression by 2.76-fold (*P* < 0.01) and promoted the phosphorylation of JAK2 and STAT5 by 1.13-fold (*P* < 0.05) and 2.82-fold (*P* < 0.01), respectively. These findings suggest that PCE promotes longitudinal bone growth in rats, potentially through GH-mediated IGF-1 regulation via the JAK2/STAT5 pathway.

## Introduction

Growth hormone (GH) and insulin-like growth factor-1 (IGF-1) are fundamental for achieving normal longitudinal bone growth and mass during the postnatal period and play major roles in bone growth and development [1]. In particular, the GH-IGF-1 axis represents a major endocrine mechanism regulating linear growth in children, with GH potently stimulating IGF-1 secretion and action in relation to the individual’s nutritional status [2,3]. GH is regulated by the interaction of GH-releasing hormone, which stimulates secretion, and somatostatin, which inhibits it [4]. The released GH binds to its receptor in the liver, activating Janus kinase 2 (JAK2), which subsequently phosphorylates signal transducer and activator of transcription 5 (STAT5). Phosphorylated STAT5 translocates to the nucleus and stimulates IGF-1 transcription [5]. Therefore, IGF-1 is closely related to GH levels and is known to play an important role in promoting bone growth and development, especially in the growth plate [6]. In fact, Lee et al. reported that the Astragalus extract mixture HT042 increased the bone growth rate by increasing IGF-1 protein levels in both the serum and liver through the phosphorylation of JAK2/STAT5 in rats, which is a normal function of the GH-dependent endocrine pathway [7]. Additionally, children with idiopathic short stature are known to have lower IGF-1 levels than healthy children [8,9].

Circulating IGF-1 is synthesized mainly in the liver as a ternary complex with IGF binding protein 3 (IGFBP3) and acid labile subunit (ALS), which prolongs the half-life of serum IGFs and promotes endocrine action. When the ternary complex reaches the growth plate through the bloodstream, it interacts with specific IGF-1 receptors located on the surface of chondrocytes to promote bone growth [10,11]. Yakar et al. crossed LID mice with ALSKO mice to generate double-knockout LID+ALSKO mice to elucidate how the reduction of IGF-1 complex affects growth and development [12]. LID+ALSKO mice presented reduced serum IGF-1 levels, whereas GH levels were markedly increased, and linear bone growth was severely impaired. These results suggest that circulating IGF-1 levels play an important role in the longitudinal bone growth rate.

Phyllostachyos Caulis in Taeniam (bamboo shaving) is the stem of bamboo [*Phyllostachys nigra* Munro var. *henonsis* Stapf, *P. bambusoides* Siebold et Zuccarini, *Bambusa tuldoides* Munro and *B. beecheyana* var. *pubescens* W.C.Lin (Gramineae)], the intermediate layer of which can be obtained after removal of the cortex. Phyllostachyos Caulis in Taeniam has been used as a traditional Chinese medicine for the treatment of fever, vomiting, hypertension and cardiovascular disease in China and the Republic of Korea and has been certified as a functional food material by the Ministry of Health in China [13–15]. Furthermore, Phyllostachyos Caulis in Taeniam extracts have been shown to have antioxidant [16], anti-inflammatory [17], neuroprotective [18], antihyperlipidemic [13,19], antihypertensive [13] and antitumor [20] effects. In addition, Zhang et al. reported that bamboo shaving extracts are safe with low toxicity and can be utilized in various foods through acute toxicity tests, mutagenicity tests, and 30-day feeding [21]. Phyllostachyos Caulis in Taeniam extract contains bioactive substances such as chlorogenic acid, caffeic acid, ferulic acid, *p*-coumaric acid, orientin, homoorientin, vitexin, and isovitexin [16], and an aqueous extract of Phyllostachyos Caulis in Taeniam (PCE) in this study contains *p*-coumaric acid as the main component. *p*-Coumaric acid is known to have excellent antioxidant effects [22]. In addition, in our previous study, *p*-coumaric acid administration increased the secretion of serum GH and IGF-1 and promoted bone growth [23]. Therefore, *p*-coumaric acid is considered an important effective ingredient in the context of this study, although further research is needed to conclusively establish it as the sole primary active component.

In our previous studies, Chung et al. reported that the total tibial length significantly increased following PCE administration, which was attributed to increases in the heights of the proliferative and hypertrophic zones in the tibia. Additionally, the ratio of BrdU-positive to total cells increased, and the serum levels of osteocalcin, GH, and IGF-1 were significantly elevated in the group treated with PCE [24]. However, the mechanisms by which PCE regulates systemic IGF-1 levels and promotes bone elongation have not yet been characterized at the molecular level. Therefore, to elucidate the mechanism of action, this study evaluated hepatic IGF-1 and IGFBP3 mRNA expression via quantitative real-time polymerase chain reaction (qRT-PCR) and hepatic IGF-1 protein levels and the phosphorylation of JAK2/STAT5 via western blotting in response to PCE administration.

## Materials and methods

### Phyllostachyos Caulis in Taeniam extract (PCE)

Dried Phyllostachyos Caulis in Taeniam was purchased from Hebei Renxin Pharmaceutical Co., Ltd. (Anguo, China), and the PCE (Batch No. T210301, T210401 and T241001) was manufactured by Zhejiang Conba Pharmaceutical Co., Ltd. (Hangzhou, China). The dried Phyllostachyos Caulis in Taeniam (400 kg) was extracted in water (10-fold dry *w/v*) at 100°C for 2 h in duplicate. The extraction liquid was cooled to below 35°C and filtered through an 800-mesh filter. The filtered liquid was concentrated via a single-effect evaporator, followed by spray drying to obtain a powdered form (average yield: approximately 5%). The obtained PCE was stored at room temperature until use and administered as an aqueous extract without additional excipients.

### High-performance liquid chromatographic analysis

We aimed to establish a standard for PCE by analyzing the *p*-coumaric acid content for consistent PCE manufacturing. High-performance liquid chromatography (HPLC) analysis of PCE and *p*-coumaric acid was conducted with an Agilent 1260 Infinity (DAD) (Agilent, SC, USA), and the analytical column used was an Agilent Eclipse plus C18, 5 μm, 250 × 4.6 mm. We used the following gradient system with 0.1% TFA/water (A) and acetonitrile (B): 0–5 min, 90% A and 10% B; 5–30 min, 75% A and 25% B; 30–33 min, 75% A and 25% B; 33–34 min, 10% A and 90% B; 34–36 min, 10% A and 90% B; 36–37 min, 90% A and 10% B; and 37–45 min, 90% A and 10% B. The flow rate was 0.9 mL/min, and *p*-coumaric acid was monitored at 320 nm. The sample injection volume was 10 μL, and the temperature of the column was maintained at 40°C. The peaks were monitored, with the peak areas corresponding to samples matching authentic *p*-coumaric acid standards. This analysis method was verified by an authorized institution (Korea Health Functional Food Institute, Seongnam, Republic of Korea), and the *p*-coumaric acid content of 3 batches of PCE (T210301, T210401 and T241001) was analyzed in triplicate.

### Animal experiments

This study was conducted using male Sprague‒Dawley (SD) rats obtained from Daehan BioLink (Eumseong, Republic of Korea). The rats were 3 weeks of age and acclimated under laboratory conditions for 7 days prior to the experiment. All the animals were housed in individually ventilated cages in a specific pathogen-free (SPF) facility, with two rats per cage, and the environmental conditions (temperature: 22 ± 2°C, relative humidity: 55 ± 10%, ventilation rate: 10–15 times per hour, light/dark cycle: 12 h, lighting: 150–200 lux) were controlled. The animals were provided with standard rodent chow and drinking water ad libitum. Both food and water were sterilized via an autoclave installed in the SPF facility before being supplied, with the water in the bottles replaced twice weekly. The rats were allocated to experimental groups via a Z score randomization method on the basis of body weight after acclimation and were divided into 4 groups (7 rats per group) for the experiment for 10 days: (1) the control (CON) group, in which 0.9% saline was administered orally (PO) at 10 mL/kg; (2) the GH group, in which recombinant human GH (rhGH, PeproTech, NJ, USA) was administered subcutaneously (SC) at 20 μg/kg/day; (3) the PCE 200 group, in which PCE (Batch No. T210301) was administered at 200 mg/kg/day; and (4) the PCE 400 group, in which PCE (Batch No. T210301) was administered at 400 mg/kg/day. GH and PCE were dissolved in 0.9% saline, the same solvent used for the control group, and then administered. At the end of the study, all the animals were deeply anesthetized with isoflurane (induction at 3–4%, maintenance at 1.5–2% in oxygen). While under deep anesthesia, a midline laparotomy was performed, and euthanasia was conducted by exsanguination via cardiac puncture. All animal experiments were conducted at CHAON (Seoul, Republic of Korea) in accordance with institutional and international guidelines for the care and use of laboratory animals and were approved by the Institutional Animal Care and Use Committee (IACUC) under protocol number CE24250, which was approved on May 3, 2024. To minimize the number of animals, a total of 28 animals were used, with 7 animals per group, and all efforts were made to minimize animal suffering.

### Tibial length measurement

For tibial length measurement, the animals were anesthetized with isoflurane (induction at 3–4%, maintenance at 1.5–2% in oxygen) and the right hind limb was carefully incised to expose the tibia. Tibial length was measured via a digital caliper, and photographs were taken for documentation. All procedures were performed under deep anesthesia to minimize pain and ensure animal welfare.

### Enzyme-linked immunosorbent assay (ELISA) analysis

Blood samples for ELISA analysis were collected at the time of necropsy. The animals were fasted for 18 h, deeply anesthetized with isoflurane, and blood samples were collected via the abdominal vena cava. The blood was anticoagulated with Na-heparin and then centrifuged at 4°C and 5,000 rpm for 10 min, after which the serum was stored at −80°C. The serum concentrations of GH, IGF-1, IGFBP3, and osteocalcin were measured via the following commercially available ELISA kits: Rat/Mouse Growth Hormone ELISA Kit (Millipore, MA, USA); Rat IGF-1 ELISA Kit (Abcam, MA, USA); Rat IGFBP-3 ELISA Kit (Elabscience, Wuhan, China); and Rat Osteocalcin ELISA Kit (LSBio, WA, USA).

### Quantitative real-time polymerase chain reaction (qRT‒PCR) analysis

Total RNA was extracted from liver tissue via the AccuPrep^®^ Universal RNA Extraction Kit (Bioneer, Daejeon, Republic of Korea) according to the manufacturer’s protocol. The RNA purity and concentration were assessed via a NanoDrop spectrophotometer. Complementary DNA (cDNA) was synthesized from 1 μg of RNA via a Labopass cDNA synthesis kit (Cosmogenetech, Seoul, Republic of Korea). Quantitative PCR was performed via TB Green^®^ Premix Ex Taq™ II (Takara, Shiga, Japan) in a QuantStudio 1 Real-Time PCR System (Applied Biosystems). The reaction conditions included initial denaturation at 95°C for 30 seconds, followed by 40 cycles of 95°C for 5 seconds and 60°C for 30 seconds. The gene expression levels of IGF-1 and IGFBP-3 were normalized to those of glyceraldehyde-3-phosphate dehydrogenase (GAPDH) and calculated via the ΔΔCt method. The sequences of the primers used were as follows [7]: IGF-1: 5’-GCTATGGCTCCAGCATTCG-3’, 5’-TCCGGAAGCAACACTCATCC-3’; IGFBP-3: 5’-GGAAAGACGACGTGCATTG-3’, 5’-GCGTATTTGAGCTCCACGTT-3’; and GAPDH: 5’-TGGCCTCCAAGGAGTAAGAAAC-3’, 5’-CAGCAACTGAGGGCCTCTCT-3’.

### Western blot analysis

Liver tissues were homogenized in RIPA buffer supplemented with protease and phosphatase inhibitors. Protein concentrations were quantified via the bicinchoninic acid (BCA) assay. Equal amounts of protein (30–50 μg) were resolved on SDS‒PAGE gels and transferred onto PVDF membranes (Bio-Rad, CA, USA). The membranes were blocked with 5% BSA in Tris-buffered saline with 0.1% Tween-20 (TBST) for 1 hour at room temperature. The membranes were subsequently incubated overnight at 4°C with primary antibodies specific for IGF-1 (Santa Cruz, CA, USA), p-JAK2 (Abcam, MA, USA), p-STAT5 (Cell Signaling, MA, USA), and corresponding internal controls (e.g., Actin, t-JAK2, and t-STAT5). After being washed with TBST, the membranes were incubated with HRP-conjugated secondary antibodies for 1 hour at room temperature. The protein bands were visualized via enhanced chemiluminescence (ECL) and quantified via ImageJ software.

### Statistical analysis

All the statistical analyses were performed via GraphPad Prism version 8.4.3 (GraphPad Software, CA, USA). The data are presented as the mean ± standard deviations (SDs). The assumption of normality was tested via the Shapiro–Wilk test, and the homogeneity of variance was assessed via Levene’s test. One-way analysis of variance (ANOVA) was conducted, followed by Dunnett’s post hoc test for multiple comparisons versus the control group. A *p*-value of less than 0.05 was considered statistically significant.

## Results

### *p*-Coumaric acid in the PCE

**Fig 1:**
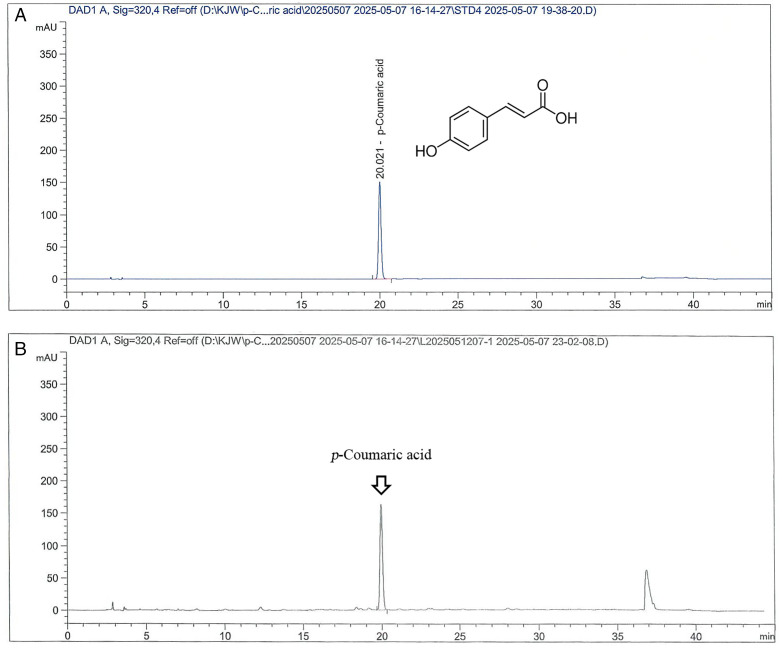
HPLC chromatograms of *p*-coumaric acid in the PCE. HPLC chromatograms of (A) *p*-coumaric acid and **(B)** PCE (Batch No. T210301).

We validated the HPLC analytical method for *p*-coumaric acid in PCE (Batch No. T241001) and confirmed the suitability of the method as shown in Table 1. Therefore, the content of *p*-coumaric acid in 3 batches of PCE was analyzed via a validated HPLC analysis method. As a result, the retention time was within the 20-minute range for both the *p*-coumaric acid standard and *p*-coumaric acid in PCE, and the content of *p*-coumaric acid in PCE (T210301) used in the animal test was 5.47 mg/g (Fig 1). Additionally, the contents of *p*-coumaric acid in the other 2 batches of PCE (T210401 and T241001) were measured as 5.30 and 4.24 mg/g, respectively.

**Table 1:** Validation of the *p*-coumaric acid analysis method in PCE.

Test items		Results
Specificity		Clear peak separation of *p*-coumaric acid was confirmed in the HPLC chromatogram, and the same spectrum and retention time (approximately 20.3 minutes) were confirmed in PCE.
Linearity		R2 = 0.99 or higher in the range of 5 concentrations (approximately 0.00 ~ 86.10 μg/mL)
Accuracy		Recovery rate (%): 98.78 ~ 104.90
Precision	Repeatability	Average content (mg/g): 4.31 ~ 4.41, RSD (%): 0.46 ~ 1.84
	Reproducibility	Average content (mg/g): 4.35 ~ 4.42, RSD (%): 0.84

### Changes in body weight and food consumption

Table 2 shows the body weight and food consumption data of the different experimental groups throughout the 10-day treatment. There was no significant difference in body weight and food consumption among the groups, and no significant differences were observed in any group (*P* > 0.05).

**Table 2:** Changes in body weight and food consumption during the 10-day treatment period.

	Group^a^	Initial	Day 5	Day 10
Body weight (g)	CON	86.7 ± 7.13	122.8 ± 8.01	161.6 ± 9.14
	GH	86.8 ± 6.60	121.0 ± 7.48	160.5 ± 7.15
	PCE200	86.8 ± 5.61	120.8 ± 7.62	158.3 ± 10.72
	PCE400	87.3 ± 4.38	123.4 ± 5.98	162.5 ± 6.91
Food consumption (g)	CON	14.7 ± 0.83	15.7 ± 0.36	18.2 ± 0.28
	GH	14.3 ± 0.05	16.4 ± 0.15	16.7 ± 1.57
	PCE200	13.5 ± 0.18	14.5 ± 0.05	17.5 ± 1.04
	PCE400	13.4 ± 0.52	17.1 ± 1.63	17.8 ± 1.38

^a^CON, control group; GH, rhGH administration group; PCE200, 200 mg/kg PCE administration group; PCE400, 400 mg/kg PCE administration group. The data are presented as the means ± SDs (n = 7). Statistical analysis was performed via two-way ANOVA followed by Dunnett’s post hoc test to compare the results with those of the control group.

### Effect on tibial length

In our previous study, we obtained histological analysis results showing that the administration of PCE and *p*-coumaric acid significantly increased the height of the proliferation and hypertrophic zones of the growth plate [23,24]. Therefore, in this study, we simply measured the tibial length for the purpose of supporting the previous results. To confirm the effect of PCE on tibial growth, we measured the tibial length of the right hind limbs excised at autopsy (Table 3). Compared with the control group (CON, 30.61 ± 0.66 mm), the rhGH-treated group (GH, 31.73 ± 1.04 mm) presented an increase of 1.12 mm (**P* *< 0.05). In addition, compared with the control group, the 200 mg/kg PCE-treated group (PCE 200, 31.96 ± 0.67 mm) and the 400 mg/kg PCE-treated group (PCE 400, 32.87 ± 0.44 mm) presented increases of 1.35 mm and 2.26 mm, respectively (*P* < 0.01), and all the administration groups presented statistically significant increases compared with the control group.

**Table 3:** Effect of PCE on tibial length in rats.

Group^a^	Tibia length (mm)
CON	30.6 ± 0.66
GH	31.7 ± 1.04*
PCE200	32.0 ± 0.67**
PCE400	32.9 ± 0.44**

^a^CON, control group; GH, rhGH administration group; PCE200, 200 mg/kg PCE administration group; PCE400, 400 mg/kg PCE administration group. The data are presented as the means ± SDs. ^*^ *P* < 0.05 and ^**^ *P* < 0.01, significantly different from the CON group (n = 7 per group). Statistical analysis was performed via one-way ANOVA followed by Dunnett’s post hoc test to compare the results with those of the control group.

### Effects on the serum GH, IGF-1, IGFBP3 and osteocalcin levels

**Fig 2:**
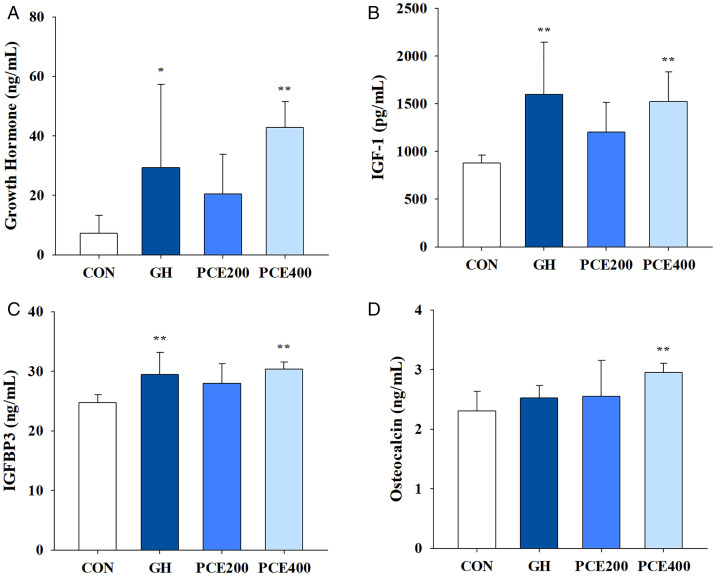
Effects of PCE on total serum (A) GH, (B) IGF-1, (C) IGFBP3, and (D) osteocalcin levels in rats. CON, control group; GH, rhGH administration group; PCE200, 200 mg/kg PCE administration group; PCE400, 400 mg/kg PCE administration group. The data are presented as the means ± SDs. Statistical analysis was performed via one-way ANOVA followed by Dunnett’s post hoc test for comparisons with CON group. ^*^ *P* < 0.05 and ^**^ *P* < 0.01, significantly different from the CON group (n = 7 per group).

To determine whether PCE administration increases the levels of serum biomarkers associated with bone growth, we measured the serum GH, IGF-1, IGFBP3, and osteocalcin levels via ELISA. Compared with the control, 400 mg/kg PCE significantly increased the serum GH, IGF-1, IGFBP3 and osteocalcin levels after 10 days of treatment (Fig 2). Specifically, the serum GH levels of the GH group (29.34 ng/mL, *P* < 0.05) and the PCE400 group (42.85 ng/mL, *P* < 0.01) were significantly different from those of the control group (7.31 ng/mL). The serum levels of IGF-1 and its complex mediator, IGFBP3, were also significantly greater in the GH (1,598 pg/mL and 29.4 ng/mL, respectively, *P* < 0.01) and PCE400 (1,521 pg/mL and 30.3 ng/mL, respectively, *P* < 0.01) groups than in the control group (876 pg/mL and 24.8 ng/mL, respectively). Finally, the serum osteocalcin levels did not significantly differ between the GH and PCE200 groups and the control group, but the serum osteocalcin level in the PCE400 group (2.95 ng/mL, *P* < 0.01) was significantly greater than that in the control group (2.31 ng/mL).

### Effects on IGF-1 and IGFBP3 mRNA expression levels

**Fig 3:**
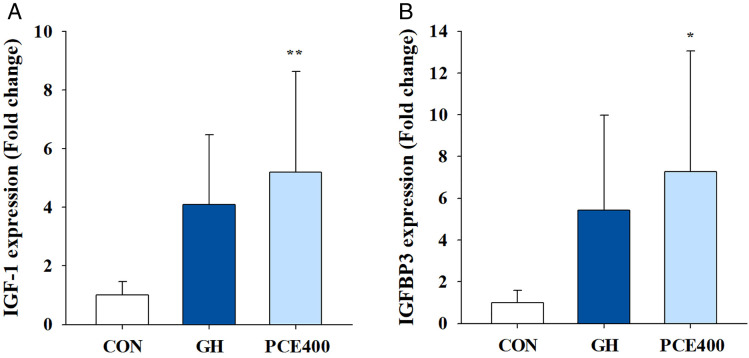
The relative expression of the IGF-1 and IGFBP3 mRNAs in the liver was measured via qRT‒PCR. Relative transcriptional levels of **(A)** IGF-1 and **(B)** IGFBP3 were analyzed via normalization to the GAPDH internal control. Molecular analyses were primarily focused on the 400 mg/kg PCE group, as the 200 mg/kg PCE group did not show significant results in initial serum biomarker analyses, and the 400 mg/kg dose aligns with ongoing clinical trial concentrations. CON, control; GH, rhGH; PCE 400, 400 mg/kg PCE. The data are presented as the means ± SDs. Statistical analysis was performed via one-way ANOVA followed by Dunnett’s post hoc test for comparisons with the CON group. ^*^ *P* < 0.05 and ^**^ *P* < 0.01, significantly different from the CON group (n = 7 per group).

Serum IGF-1 is synthesized mainly in the liver in a GH-dependent manner, so qRT‒PCR was conducted to measure liver IGF-1 and IGFBP3 mRNA expression [25]. In addition, to determine whether PCE intake affects IGF-1 and IGFBP3 expression, only the 400 mg/kg dose among the PCE administration groups was analyzed. As shown in Fig 3, the rhGH-treated group presented increased levels of the IGF-1 and IGFBP3 mRNA in the liver, but these differences were not statistically significant compared with those of the control group. In contrast, compared with the control group, the 400 mg/kg PCE group presented a significant 5.2-fold increase in the mRNA expression of IGF-1 (*P* < 0.01) and a significant 7.3-fold increase in the expression of IGFBP3 (*P* < 0.05) in the liver.

### Effects on IGF-1 and regulatory proteins

**Fig 4:**
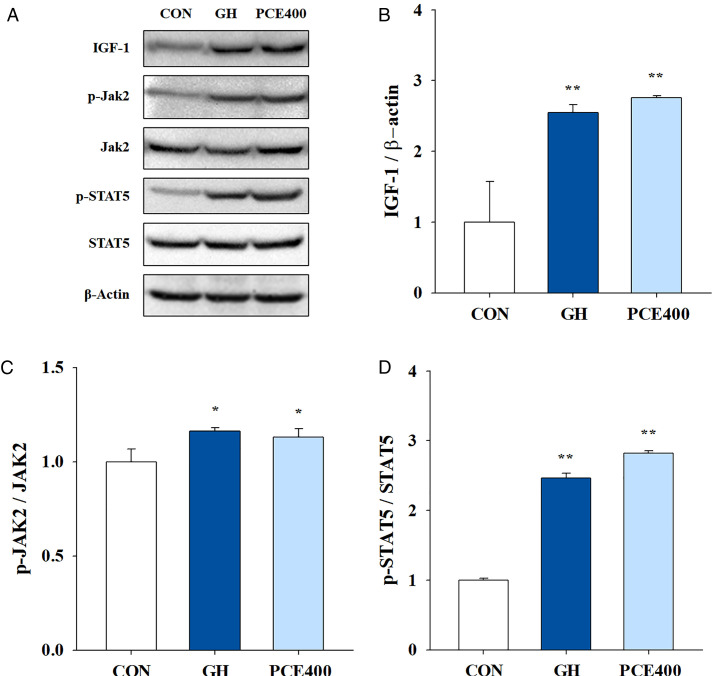
The protein levels of IGF-1, JAK2 and STAT5 in the liver were measured via western blotting. Molecular analyses were primarily focused on the 400 mg/kg PCE group, as the 200 mg/kg PCE group did not show significant results in initial serum biomarker analyses, and the 400 mg/kg dose aligns with ongoing clinical trial concentrations. **(A)** Representative western blots of the indicated proteins, **(B)** IGF-1, (C) phosphor- **(*p*-)**JAK2/total- **(t-)**JAK2, and (D) *p*-STAT5/t-STAT5. CON, control; GH, rhGH; PCE 400, 400 mg/kg PCE. The data are presented as the means ± SDs. Statistical analysis was performed via one-way ANOVA followed by Dunnett’s post hoc test for comparisons with the CON group. ^*^ *P* < 0.05 and ^**^ *P* < 0.01, significantly different from the CON group (n = 3 per group).

To determine whether the growth-stimulating effect of PCE was mediated by the phosphorylation of JAK2 and STAT5, which induce IGF-1 production, Western blotting was performed on liver tissue. As a result, the IGF-1 protein was significantly expressed in the GH (2.55-fold) and PCE400 (2.76-fold) groups compared with the control group (*P* < 0.01), and the phosphorylation of JAK2 and STAT5 was also increased by the administration of rhGH and PCE (Fig 4). In particular, compared with total JAK2 and STAT5, phosphorylated JAK2 increased 1.16-fold in the GH group (*P* < 0.05) and 1.13-fold in the PCE400 group (*P* < 0.05), and phosphorylated STAT5 increased 2.47-fold in the GH group (*P* < 0.01) and 2.82-fold in the PCE400 group (*P* < 0.01).

## Discussion

To determine the effects of PCE on bone growth, we evaluated tibial length, biochemical and molecular growth-related factors in an experimental adolescent rat model and found significant differences in these factors compared with those in the control group. In this study, we administered PCE to 4-week-old adolescent rats for 10 days. This decision was made on the basis of previous reports that rats begin to grow and develop at 30 days of age [26] and previous papers that reported significant results in terms of bone growth after 10 days of administration [7,23,24]. According to the results of this study, the rats in the PCE administration group presented changes in tibial length after 10 days of treatment. In addition, PCE administration significantly increased the total serum concentrations of GH, IGF-1, IGFBP3, and osteocalcin and significantly increased the expression of IGF-1 and IGFBP3 mRNAs in the liver. Finally, PCE administration increased the protein expression of IGF-1 in the liver, possibly through the promotion of JAK2 and STAT5 phosphorylation. Taken together, these results suggest that PCE may increase IGF-1 production through promoting the phosphorylation of JAK2 and STAT5 in the liver, thereby promoting longitudinal bone growth.

Phyllostachyos Caulis in Taeniam (bamboo shaving) is the middle layer of bamboo, with the outer bark removed. It has been traditionally used in the Republic of Korea and China to reduce fever and stop internal bleeding and is called Bambusae Caulis in Taeniam (Zhuru) in China. In our previous study, X-ray and micro-CT analyses revealed that PCE had excellent effects on bone growth and significantly increased the secretion of serum GH, IGF-1, and osteocalcin, as determined via ELISA [24]. In addition, we reported in our previous study that there was no change in the weights of the spleen, kidney, heart, lung, brain, and liver when PCE was repeatedly administered to rats at concentrations of up to 5 g/kg/day for 13 weeks [24]. These findings are consistent with those of a previous study in which bamboo shaving extract was shown to be highly safe in rats [21]. The PCE used in this study was standardized through content analysis of *p*-coumaric acid, which was verified by an authorized institution in the Republic of Korea. We manufactured 3 batches of PCE in a GMP facility and analyzed the *p*-coumaric acid content of each batch. The average content was 5 mg/g, establishing a standard of 4–6 mg/g, which is approximately 20%. *p*-Coumaric acid is a major component of the PCE, and in our previous study, it increased bone growth and the secretion of serum GH and IGF-1 [23]. On the basis of these results, we established *p*-coumaric acid as a marker compound and biologically active compound of PCE.

To support the results of previous studies, we aimed to elucidate the mechanism by which PCE, an aqueous extract of Phyllostachyos Caulis in Taeniam, secretes serum markers related to bone growth efficacy. First, there was no significant change in body weight or feed intake among the experimental groups over 10 days, suggesting that PCE may not affect the appetite of the animals. In addition, to confirm whether the results were equivalent to those of previous studies and in terms of bone growth, tibial length was measured by removing the right hind limb at autopsy. The proximal tibial epiphysis is closely related to the growth plate, and the growth of the tibial epiphysis determines the final tibial length [27]. Similar to the results of previous studies, tibial length significantly increased in the groups administered rhGH and PCE compared with the control group.

IGF-1 is known as an important regulator of bone metabolism and a stimulator of bone formation and acts as a regulator of bone resorption [28]. One of the direct actions of GH is to stimulate IGF-1 in the liver [29]; therefore, serum GH and IGF-1 levels can be used as very important indicators of bone growth. In this study, the concentrations of serum GH and IGF-1 were significantly greater in the rhGH and PCE 400 mg/kg groups than in the control group. In serum, the majority of IGFs exist in a 150-kDa complex composed of one IGF molecule, IGFBP3, and the acid labile subunit (ALS) [30]. Therefore, the serum IGFBP3 concentration is also very important, and the administration of rhGH and PCE significantly increased the concentration of serum IGFBP3. Osteocalcin is a noncollagenous protein produced by osteoblasts that circulates in the blood. The secretion of osteocalcin is proportional to bone turnover; therefore, the serum osteocalcin level can be used as a parameter of bone formation [31]. As observed in previous studies, PCE administration significantly increased osteocalcin levels, suggesting that PCE may contribute to the activation of osteoblasts.

Circulating IGF-1 is mostly synthesized in the liver in a GH-dependent manner, and approximately 80% of circulating IGF-1 is bound to IGFBP3, which prolongs its half-life and is transported to the receptor [32]. Therefore, in this study, we aimed to determine whether PCE administration increases the mRNA expression of IGF-1 and IGFBP3 in rat liver tissue, and we were able to observe greater increases in the expression of these genes in the PCE-treated group than in the control group.

GH signal transduction is mediated through protein phosphorylation cascades to activate nuclear proteins and transcription factors [33]. When GH binds to a receptor, JAK2 is activated, and STAT5 is subsequently phosphorylated. STAT5 translocates to the nucleus and binds to specific DNA sequences to regulate transcription [34], and this process mainly stimulates IGF-1. Therefore, among the signal transduction pathways induced by GH, the JAK2 and STAT5 signaling pathways are considered to play important roles in IGF-1 production in the liver [35]. In this study, compared with the control, PCE promoted the phosphorylation of JAK2 and STAT5 and significantly increased the protein expression of IGF-1 in the liver. Our finding that PCE increases the phosphorylation of JAK2 and STAT5 as well as the IGF-1 levels in the serum and liver suggests that the growth-stimulating effect of PCE is mediated by an increase in circulating IGF-1 via the JAK2 and STAT5 signaling pathways.

This study is limited by its reliance on a rat model, which may not fully translate to human physiology without further clinical validation. The short 10-day treatment period restricts insights into the long-term efficacy and safety of PCE. In addition, while our previous study performed histological bone analysis, this study lacked histological bone analysis, had a narrow animal age range of 4–5 weeks, and was limited to male rats. Studies focusing on the JAK2/STAT5 pathway have not explored other potentially relevant mechanisms or broader metabolic effects. While significant effects were primarily observed at the higher PCE dose (400 mg/kg) and molecular analyses were conducted at this dose to elucidate the mechanism, future studies could further explore the full dose-response relationship at the molecular level to optimize therapeutic regimens. Additionally, while western blots were performed with n = 3 per group, potentially limiting the statistical power for these specific analyses, these results are consistent with broader physiological and gene expression findings, and future studies will aim to increase the number of replicates for molecular analyses to increase statistical confidence.

In conclusion, this study demonstrated that PCE has the potential to stimulate the rate of bone growth by increasing hepatic IGF-1 and IGFBP3 mRNA levels and increasing serum and liver IGF-1 protein levels through JAK2/STAT5 phosphorylation. On the basis of these study results, further clinical studies, such as those assessing children’s height growth, are needed to prove the potential value of PCE.
