# Correction: Phyllostachyos Caulis in Taeniam extract stimulates longitudinal bone growth via IGF-1 and JAK2/STAT5 signaling in rats

**DOI:** 10.1371/journal.pone.0339511

**Published:** 2025-12-22

**Authors:** The PLOS One Staff

There are errors in the author affiliations. The correct affiliations are as follows:

Yongwun An^1,2,3^, Hee Woo Lee^4^, Yun Chan Jung^4^, Hyun Jae Woo^4^, Younghoon Kim^4^, Hohyun Kim^2^, Yong Joon Jeong^2^, A. M. Abd EI-Aty^5,6^, Ji Hoon Jeong^1,3^

**1** Department of Pharmacology, College of Medicine, Chung-Ang University, Seoul, Republic of Korea, **2** Korea Medicine Research Institute, Inc., Seongnam, Republic of Korea, **3** Department of Global Innovative Drugs, Graduate School of Chung-Ang University, Seoul, Republic of Korea, **4** Chaon Co., Ltd., Yongin, Republic of Korea, **5** Department of Pharmacology, Faculty of Veterinary Medicine, Cairo University, Giza, Egypt, **6** Department of Medical Pharmacology, Medical Faculty, Ataturk University, Erzurum, Turkey.

The publisher apologizes for the error.
